# Changing Incidence and Characteristics of Photokeratoconjunctivitis During the COVID-19 Pandemic

**DOI:** 10.5811/westjem.17882

**Published:** 2024-04-09

**Authors:** Yu-Shiuan Lin, Chih-Cheng Lai, Yu-Chang Liu, Shu-Chun Kuo, Shih-Bin Su

**Affiliations:** * Chi Mei Medical Center, Department of Ophthalmology, Tainan, Taiwan; † Chi Mei Medical Center, Division of Hospital Medicine, Department of Internal Medicine, Tainan, Taiwan; ‡ Chi Mei Medical Center, Department of Emergency Medicine, Tainan, Taiwan; § National Cheng Kung University, College of Medicine, Department of Environmental and Occupational Health, Tainan, Taiwan; ∥ Chung Hwa University of Medical Technology, Department of Optometry, Jen-Teh, Tainan, Taiwan; ¶ Chi Mei Medical Center, Department of Occupational Medicine, Tainan, Taiwan; # Chi Mei Medical Center, Department of Family Medicine, Tainan, Taiwan

**Keywords:** COVID-19, SARS-CoV-2, ultraviolet light, photokeratoconjunctivitis, germicidal lamp, welding

## Abstract

**Introduction:**

Photokeratoconjunctivitis (PKC) is primarily caused by welding. However, inappropriate use of germicidal lamps, which have been widely used following the COVID-19 outbreak, can also cause PKC. Our goal in this study was to investigate the incidence of and changes in the causes of PKC during the coronavirus 2019 (COVID-19) pandemic.

**Methods:**

We conducted a single-center, retrospective observational study. The health records of patients who visited the emergency department in a tertiary care hospital from January 1, 2018–December 31, 2021 and were diagnosed with PKC, were reviewed. We then conducted an analysis to compare the characteristics of PKC before and after COVID-19 began and the features of PKC caused by welding and germicidal lamps.

**Results:**

There were 160 PKC cases with a clear etiology before the COVID-19 pandemic and 147 cases during the COVID-19 pandemic. No significant differences in age and gender were detected between the two groups. The incidence of PKC induced by the use of germicidal lamps during the COVID-19 pandemic was significantly higher (10.2%) than the incidence before the pandemic (3.1%). The ratio of females to males in the germicidal lamp subgroup was significantly higher than the ratio in the welding subgroup. Limitations included incomplete information due to the retrospective nature of the study, underestimation of incidence, and possible recall bias.

**Conclusion:**

In the era of COVID-19, clinicians should be aware of the hazards of germicidal lamps. Although the COVID-19 pandemic seems to show signs of easing, new infectious diseases that require protective measures could still emerge in the future. Therefore, injuries related to germicidal lamps deserve more public health attention.

Population Health Research Capsule What do we already know about this issue?

*Photokeratoconjunctivitis (PKC) is mainly caused by welding. Germicidal lamps, which also cause PKC if improperly used, were widely used after the COVID-19 outbreak.*

What was the research question?

*What are the incidence of and changes in the causes of PKC during the COVID-19 pandemic (over a two-year period)?*

What was the major finding of the study?

*The incidence of PKC induced by use of germicidal lamps increased significantly after COVID-19 began (10.2% vs 3.1%).*

How does this improve population health?

*The potential injuries from germicidal lamps deserve more public health attention both during the COVID-19 era and for new infectious diseases in the future.*

## INTRODUCTION

Photokeratoconjunctivitis (PKC), or photophthalmia, is related to ultraviolet radiation (UVR) exposure. Exposure to ultraviolet B (UV-B) and ultraviolet C (UV-C) can damage the ocular surface, including the corneal or conjunctival epithelial cells.^1^ The clinical manifestations include ocular pain or foreign body sensation, tearing, photophobia, and even blurred vision in severe cases. The photochemical reaction typically takes 6–12 hours to cause symptoms.^2^ Therefore, patients often experience symptoms at night after daytime exposure, leading to emergency department (ED) visits at night.^3^ Exposure to UVR can be classified into natural and artificial sources. Natural sources include direct or reflected sunlight during skiing or time spent at the beach.^4^ Artificial sources include workplace welding flashes, which are the most common cause of PKC, and curing lights, printing machines, high-tech industrial processes, laser engravers, and germicidal lamps.^3^

The coronavirus disease 2019 (COVID-19) is caused by the severe acute respiratory syndrome coronavirus 2 (SARS-CoV-2). The COVID-19 outbreak began in December 2019 and was declared a pandemic by the World Health Organization in 2020.^5^^,^^6^ To control the spread of this highly contagious virus, several preventative and control measures were implemented, including wearing masks, social distancing, hand hygiene, and use of personal protective equipment and disinfectants, such as diluted bleach solutions or 70% ethanol.^6^^,^^7^ Ultraviolent radiation was investigated for irradiation of the coronavirus^8^; UVR disinfects by damaging DNA structures, including viral DNA. Different wavelengths have different disinfecting effects.^9^ The potential hazard of germicidal lamps to the eyes was recognized before the COVID-19 pandemic.^10^^,^^11^ However, germicidal lamp use increased significantly during the COVID-19 pandemic, and the increased usage may lead to more cases of PKC.

Changes in ophthalmic ED visits after the COVID-19 pandemic began were recently discussed. A decreased number of overall eye injuries was noted in several studies.^12^^,^^13^ Additionally, several studies reported cases of PKC due to germicidal lamps after the COVID-19 pandemic began, and an eight-week comparison study suggested an upward trend.^14^^–^^16^ However, long-term data about the causes of PKC after the COVID-19 pandemic is limited. In this study we aimed to investigate the incidence of and changes in the causes of PKC before and after the COVID-19 pandemic.

## METHODS

### Study Design

We divided patients into two groups: patients with PKC before the COVID-19 pandemic (between January 1, 2018–December 31, 2019) and patients with PKC after the COVID-19 pandemic began (between January 1, 2020–December 31, 2021). Demographic data, including gender, age, month, time, etiology, occupation, and exposure time, were collected.

### Participants

Patients who visited the tertiary-care center ED in Taiwan between January 1, 2018–December 31, 2021 and were diagnosed with PKC were enrolled in the study. Photokeratoconjunctivitis was diagnosed according to the following criteria: UV exposure history within one day; typical symptoms, including ocular foreign body sensation, pain, photophobia, and tearing; and ophthalmic clinical findings, such as conjunctival hyperemia and corneal superficial punctate lesions. We excluded pediatric patients, given the possibility of uncooperative physical and ocular examination. Based on the causes of PKC mentioned in the medical records, patients were divided into three groups based on the etiology: germicidal lamps; welding; and other. Patients whose medical records specifically mentioned the use of germicidal lamps were assigned to the germicidal lamp group, patients whose medical records mentioned exposure to welding were assigned to the welding group, and those whose causes were unknown or related to direct or reflective sunlight were assigned to the others group. This study was approved by the Institutional Review Board of Chi Mei Medical Center, Tainan, Taiwan (Applicant’s No: 11108-010).

### Outcome Measures

The primary outcome of the study was the incidence and causes of PKC before and after COVID-19 began. The secondary outcome was the characteristics of the germicidal lamp and welding groups.

### Statistical Analysis

We analyzed data using SPSS Statistics for Windows, version 22 (IBM Corp, Armonk, NY). The Pearson chi-squared test and Fisher exact test were used to compare categorical variables. Continuous variables were compared using the Student *t*-test. The threshold for statistical significance was defined as a *P*-value less than 0.05.

## RESULTS

### Patient Characteristics

During the study period, 307 PKC patients were recruited. Fewer cases of PKC occurred after COVID-19 began (147 vs 160) compared with the number of PKC cases before the COVID-19 period. The mean patient ages were 41.85 ± 0.97 years (range, 20–71 years) before COVID-19 and 40.07 ± 1.09 years (range, 20–78 years) after COVID-19. No significant differences in age or gender were detected between the groups before and after COVID-19 began. A majority of patients went to the ED at night; 90% went to the ED between 8 pm–07:59 am, and the most prevalent period was 12 am–03:59 pm. The characteristics of the patients with PKC are summarized in Table 1.

**Table 1.** Characteristics of patients with photokeratoconjunctivitis.

	Before COVID-19 (N = 160)	After COVID-19 (N = 147)	*P* value
Age: mean ± SD	41.85 ± 0.97	40.07 ± 1.09	0.22
Gender (%)			0.88
Male	154 (96.3)	141 (95.9)	
Etiology of PKC (%)			*P* = 0.03
Welding	144 (90.0)	118 (80.3)	
Germicidal lamp	5 (3.1)	15 (10.2)	
Other causes	11 (6.9)	14 (9.5)	
Time of ED visit (%)			0.89
00:00–03:59	100 (62.5)	93 (63.3)	
04:00–07:59	18 (11.3)	16 (10.9)	
08:00–11:59	2 (1.3)	2 (1.4)	
12:00–15:59	1 (0.6)	2 (1.4)	
16:00–19:59	2 (1.2)	3 (2.0)	
20:00–23:59	37 (23.1)	31 (21.1)	

*COVID-19*, coronavirus disease 2019; *ED*, emergency department; *PKC*, photokeratoconjunctivitis.

### Incidence and Demographic Data of Germicidal Lamp-related PKC

The total number of PKC cases slightly decreased after the COVID-19 pandemic began. The etiologies of PKC were different before and after COVID-19 began. The percentage of patients in the germicidal lamp group before COVID-19 (5, 3.1%) was lower than the percentage of patients in the germicidal lamp group after COVID-19 began (15, 10.2%); thus, the incidence of PKC in the germicidal lamp group increased significantly after COVID-19 began (*P* = 0.03). Within the germicidal lamp-related PKC subgroup, the mean ages were 39.20 ± 3.69 years (range, 31–51 years) and 42.73 ± 3.88 years (range, 21–78 years) before and after COVID-19 began, respectively, and no significant differences in age or gender were detected between the groups (Table 2). All patients in the germicidal lamp group went to the ED between 8 pm–07:59 am.

**Table 2.** Demographic data of patients with germicidal lamp-induced photokeratoconjunctivitis before and after COVID-19 began.

	Before COVID-19 (n = 5)	After COVID-19 (n = 15)	*P* value
Age: mean ± SD (range)	39.20 ± 3.69 (31–51)	42.73 ± 3.88 (21–78)	0.62
Gender (%)			0.35^*^
Male	2 (40.0)	10 (66.7)	
Time of ED visit (%)			0.51
00:00–03:59	2 (40)	10 (66.67)	
04:00–07:59	1 (20)	1 (6.67)	
08:00–11:59	0 (0)	0 (0)	
12:00–15:59	0 (0)	0 (0)	
16:00–19:59	0 (0)	0 (0)	
20:00–23:59	2 (40)	4 (26.67)	

^*^ Fisher exact test.

*COVID-19*, coronavirus disease 19; *ED*, emergency department; *PKC*, photokeratoconjunctivitis.

### Comparison Between the Welding and Germicidal Lamp Groups

Most patients with PKC were males (more than 90% before and after COVID-19 began), and most patients in the welding subgroup were males (Tables 1 and 3). However, the percentage of females in the germicidal lamp subgroup was higher than the percentage of females in the welding group (*P* < 0.001). The times patients went to ED were not significantly different between the germicidal lamp group and the welding group.

**Table 3.** The comparison between the photokeratoconjunctivitis subgroups of germicidal lamp and welding.

	Germicidal lamp (n = 20)	Welding (n = 262)	*P* value
Age: mean ± SD (range)	41.85 ± 3.03 (21–78)	40.87 ± 0.79 (20–72)	0.74
Gender (%)			*P* < 0.001
Male	12 (60.0%)	261 (99.6%)	

### Information About Individuals in the Germicidal Lamp Group

Information about patients in the germicidal lamp group is presented in Supplemental Tables 1 and 2). The exposure duration ranged from a few seconds to two hours. The domestic component accounted for 60% (3/5) of the PKC cases before COVID-19 and 40% (6/15) of the cases after COVID-19 began. Before COVID-19, the occupational component included medical personnel/staff (2/5). After COVID-19 began, the occupational component expanded to medical staff (3/15), workers in the restaurant and hotel industry (3/15), a cleaner (1/15), a school employee (1/15), and a construction industry worker (1/15).

## DISCUSSION

In this study we compared the incidence and causes of PKC before and after COVID-19 began. We selected January 1, 2020, as the starting point for the observation period for two primary reasons. The first reason was the geographical proximity between Taiwan and Mainland China and the frequent business trips between the two nations. Secondly, based on the previous painful experience of the SARS outbreak in Taiwan in 2003, our government and people treated this incident with great caution at a very early stage. At a press conference on December 31, 2019, the Taiwan Ministry of Health and Welfare, announced epidemic information and initiation of a border quarantine in accordance with standard procedure.

The proportion of germicidal lamp-related PKC cases significantly increased after COVID-19 began. The increase in PKC cases is attributed to the increased number of germicidal lamp-related PKC cases and the decreased number of total PKC cases. The number of germicidal lamp-related PKC cases likely increased after COVID-19 began due to the increased use of these lamps for disinfection. Previous studies concerning germicidal lamp-related PKC cases after COVID-19 began are shown in Table 4. Leung reported three cases (six eyes) in Hong Kong and Sengillo reported seven cases (14 eyes) in the United States. Wang et al compared germicidal lamp-related PKC cases eight weeks before and eight weeks after COVID-19 began and reported the percentage of PKC due to disinfection increased significantly from 9.1% to 56.9% after COVID-19 began.^14^ Wang et al also mentioned that the case number decreased substantially after good public health education. In Taiwan, germicidal lamps had product instructions and warnings about improper use. The news media also emphasized the hazards of germicidal lamps. Despite the spread of public education on the use of germicidal lamps, some patients were unaware of the dangers. However, public education may have prevented upward spikes in the incidence of PKC.

**Table 4.** Summary of recent studies about germicidal lamp-induced photokeratitis after COVID-19 pandemic began.

Reference	Study population	Study design	Mean age	Gender	UV lamp type	Exposure time	Initial visual acuity	Final VA
Wang 2021^14^	109 cases in China	Retrospective	32.1 (range, 21–54)	M:F = 55:54	No record	Average: 16.7 minutes	0.25 ± 0.08 logMAR	0.05 ± 0.02 logMAR
Sengillo 2021^15^	7 cases (14 eyes) in USA	Case series	40 (range, 24–59)	M:F = 5:2	P3: 38 W UV-C germicidal lamp (AURA) P6: 38W UV-C germicidal lamp (Uvlizer)	10 minute- 4 hours in 5 cases, 2 without documentation	20/30 or better in 13/14 eyes (93%)	No record
Leung 2021^16^	3 cases (6 eyes) in Hong Kong	Case report	One is 17; no record about other two patients	M:F = 1:2	UV-C Effective illumination area: 40 m^2^	15, 20, 60 minutes	All 3 were 6/12 bilaterally	All 3 were 6/6 bilaterally
Lin 2022	15 cases in Taiwan	Retrospective	42.7 (range, 21–78)	M:F = 10:5	No record	few seconds to 2 hours, 5 cases without documentation	No record	No record

*UV*, ultraviolet; *VA*, visual acuity; *M:F*, male to female; *logMAR*; logarithm of the minimum angle of resolution.

Our study also showed that the number of total PKC (160 before; 147 after) patients and the number of patients in the welding subgroup (144 before; 118 after) slightly decreased after COVID-19 began. There are several explanations for this decrease. First, patients did not go to the ED due to concerns about SARS-CoV-2.^17^^,^^18^ Second, the outbreak forced many companies and factories to halt production (lockdown), which predominantly influenced short-term or part-time workers. According to a study by Yen et al,^3^ long-term workers complied with safety regulations better than short-term workers and wore protective equipment more. Therefore, the welding cases decreased, and germicidal lamp-related PKC cases increased, leading to the significantly increased proportion of germicidal lamp-related PKC cases after COVID-19 began.

The domestic component in the germicidal lamp subgroup declined from 60% (3/5) before COVID-19 to 40% (6/15) after COVID-19 began. In contrast, workplace cases increased from 40% (2/5) before COVID-19 to 60% after COVID-19 (9/15) began. This finding implies that hospitals/clinics/nursing homes, restaurants, and hotels required more germicidal lamp use. In Taiwan, many hotels served as quarantine hotels, where germicidal lamps were frequently used for disinfection. In Leung’s report, the three cases belonged to clustering at home.^16^ In the study of Wang et al,^14^ clustering played an important role. Our cases of germicidal lamp-related PKC were all sporadic rather than clustering episodes, which may indicate good public policies and staff safety education in most companies and workplaces.

There are some differences between welding-associated and germicidal lamp-related PKC. The wavelength emanating from germicidal lamps is mostly UV-C (254 nanometers), and the cornea, which absorbs most of the UV-C, is predominantly damaged.^9^^,^^19^^,^^20^ The wavelength emanating from welding equipment is in the UV-B spectrum. Theoretically, the energy of UV-C is greater than the energy from UV-B and, therefore, causes more damage to the cornea at the same distance and exposure time. However, most previous studies reported visual acuity as a good prognosis in germicidal lamp-related PKC cases (Table 4 **)**. Because it is primarily men who work in the welding industry, the proportion of females in the germicidal-lamp group was significantly higher than the females in the welding group in our study and previous studies.^14^

## LIMITATIONS

There were some limitations to our study. Due to its retrospective nature, some data (such as exposure time, visual acuity, brand, and wavelength of the machine) was incomplete. However, this missing data did not affect our results. Second, we may have underestimated the incidence of germicidal lamp-related PKC. Our study focused on the adult population; so pediatric patients should be taken into account in future research. In addition, we did not analyze patients without a clear PKC cause to maintain the rigor of our study. Thus, there might be recall bias. In our clinical experience, patients denied UVR or any other strong light exposure until they were specifically asked about exposure to germicidal lamps or UV-enabled dish dryers. If the medical staff did not directly ask patients about their exposure to certain machines, the exact cause of the PKC was difficult to determine. Third, the results may not be generalized to other nations because of differences in race, culture, education status, pandemic severity, and accessibility to UVR machines. Despite these limitations, we hope our study focuses more public attention on the related issues and potential hazards. The effects of news media and public safety education on the trend of germicidal lamp-related PKC after 2022 may require further studies to evaluate.

## CONCLUSION

Germicidal lamp-related PKC increased during the COVID-19 era. We found that the incidence increased significantly over a two-year period from 3.1% before COVID-19 to 10.2% after COVID-19 began. While it appears that the COVID-19 pandemic is gradually subsiding, it is important to recognize that new infectious diseases may emerge in the future, necessitating protective measures. Therefore, clinicians should pay attention to this potential cause of PKC and take more accurate histories. The potential hazard of germicidal lamps is an important public health issue that should be emphasized to prevent further injury from this source of ultraviolet radiation.

## Supplementary Information




