# Supplementary material for: Changing Incidence and Characteristics of Photokeratoconjunctivitis During the COVID-19 Pandemic
Source: West J Emerg Med. 2024 Apr 9;25(3):368–73. doi: 10.5811/westjem.17882 (PMC11112667; doi:10.5811/westjem.17882)
Supplement: Supplementary file 1 [file wjem-25-368-s001.docx]

Supplement Table 1. The characteristics of germicidal lamp-induced PKC cases before COVID-19.

| **Case** | **Year** | **Month** | **Sex** | **Age** | **Visual acuity** | **Occupation/Domestic** | **Exposure time** |
| --- | --- | --- | --- | --- | --- | --- | --- |
| 1 | 2018 | 2 | M | 31 | nil | Home | No record |
| 2 | 2018 | 4 | F | 39 | 0.8/0.8 | Hospital staff | No record |
| 3 | 2018 | 6 | F | 32 | 0.8/0.8 | Home | 15 minutes |
| 4 | 2018 | 7 | M | 51 | 0.2/0.2 | Home | No record |
| 5 | 2019 | 3 | F | 43 | nil | Clinic personnel | No record |

Supplement Table 2. The characteristics of germicidal lamp-induced PKC cases after COVID-19.

| **Case** | **Year** | **Month** | **Sex** | **Age** | **Visual acuity** | **Occupation/ Domestic** | **Exposure time** |
| --- | --- | --- | --- | --- | --- | --- | --- |
| 1 | 2020 | 1 | M | 78 | nil | Home | 1 hour |
| 2 | 2020 | 3 | M | 24 | nil | Restaurant& Hotel industry | <1 minute |
| 3 | 2020 | 6 | M | 49 | nil | Home | Few seconds |
| 4 | 2020 | 7 | F | 52 | nil | Cleaner | 5–6 minutes |
| 5 | 2020 | 11 | F | 30 | nil | Nurse | No record |
| 6 | 2020 | 11 | M | 41 | nil | Nurse | 5 minutes |
| 7 | 2021 | 1 | F | 37 | 0.6/0.8 | Restaurant& Hotel industry | Few minutes |
| 8 | 2021 | 2 | M | 58 | 0.9/1.2 | Teacher | No record |
| 9 | 2021 | 6 | M | 46 | nil | Home | 30 minutes |
| 10 | 2021 | 7 | M | 21 | 0.8/0.7 | Home | 15 minutes |
| 11 | 2021 | 7 | M | 46 | nil | Home | No record |
| 12 | 2021 | 8 | M | 55 | nil | Construction industry | No record |
| 13 | 2021 | 9 | M | 39 | nil | Restaurant& Hotel industry | 2 hours |
| 14 | 2021 | 11 | F | 24 | nil | Hospital (assistant) | 5 minutes |
| 15 | 2021 | 12 | F | 41 | nil | Home | No record |
